# Fengycin induces ion channels in lipid bilayers mimicking target fungal cell membranes

**DOI:** 10.1038/s41598-019-52551-5

**Published:** 2019-11-05

**Authors:** Anastasiia A. Zakharova, Svetlana S. Efimova, Valery V. Malev, Olga S. Ostroumova

**Affiliations:** 1 Institute of Cytology of the Russian Academy of Sciences, St. Petersburg, 194064 Russian Federation; 2 St. Petersburg State University, Petergof, 198504 Russian Federation

**Keywords:** Single-molecule biophysics, Ion transport

## Abstract

The one-sided addition of fengycin (FE) to planar lipid bilayers mimicking target fungal cell membranes up to 0.1 to 0.5 μM in the membrane bathing solution leads to the formation of well-defined and well-reproducible single-ion channels of various conductances in the picosiemens range. FE channels were characterized by asymmetric conductance-voltage characteristic. Membranes treated with FE showed nonideal cationic selectivity in potassium chloride bathing solutions. The membrane conductance induced by FE increased with the second power of the lipopeptide aqueous concentration, suggesting that at least FE dimers are involved in the formation of conductive subunits. The pore formation ability of FE was not distinctly affected by the molecular shape of membrane lipids but strongly depended on the presence of negatively charged species in the bilayer. FE channels were characterized by weakly pronounced voltage gating. Small molecules known to modify the transmembrane distribution of electrical potential and the lateral pressure profile were used to modulate the channel-forming activity of FE. The observed effects of membrane modifiers were attributed to changes in lipid packing and lipopeptide oligomerization in the membrane.

## Introduction

**Figure 1:**
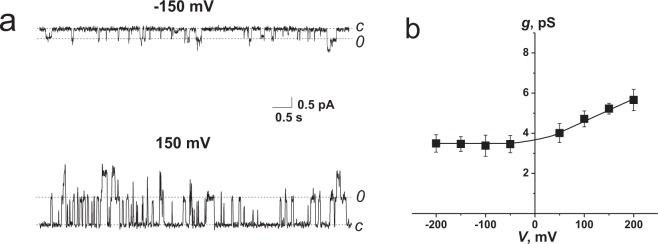
(**a**) Current fluctuations of the single FE channels at transmembrane voltages of +150 mV and −150 mV. *C* – closed state of the channel (0 pA), *O* – opened state of the pore. (**b**) Conductance–voltage curves of the single FE channels. The membrane was composed of POPC:POPE:POPG:ERG (20:20:50:10 mol%) and bathed in 2 M KCl and 10 mM CHES, pH 9. The lipopeptide concentration was equal to 0.5 μM.

Based on the developmental progress of cyclic lipopeptide daptomycin (CUBICIN®, Cubist), a trend toward finding new lipopeptide-based drugs has appeared. Compounds that directly affect the target cell membrane are most pertinent to the development of antibiotic resistance by infectious microorganisms^1^. Lipopeptide fengycin, the antifungal and antitumor agent produced by *Bacillus subtilis*, has a hemolytic activity that is 40-fold lower than that of the other well-known *Bacillus subtilis* lipopeptide surfactin^2,3^. The FE structure is composed of a β-hydroxy fatty acid linked to a peptide region comprising ten amino acids, eight of which form a lactone ring structure. The peptide portion of the lipopeptide molecule exists in four isoforms. FE and plipastin differ by *L*- and *D*-Tyr at the 3 position, respectively. FE A includes *D*-Ala at the 6-position, which is replaced by *D*-Val in the case of FE B (Fig. 1S, Supplementary Information). The lipopeptide lipid moiety can be either saturated or unsaturated and varies in length from 14 to 18 carbon atoms^4,5^. An isoform containing a β-hydroxy fatty acid with a chain length of 17 carbon atoms containing one double bond is most often found^6^.

Although the antifungal properties of FE have been well characterized^7–10^, its detailed mechanism of action is not fully understood and is a subject of intensive research. The lipopeptide produced by *Bacillus subtilis* likely acts by making the plasma membrane of the target cell more permeable^11^. Two general models have been proposed to explain lipopeptide membrane activity: functioning in a detergent-like manner via solubilization of the lipid bilayer^12,13^ and formation of transmembrane pores that lead to permeability changes^14,15^.

While the «detergent» hypothesis predominates over that of the «pore»^16,17^, it fails to explain the activity of FEs against specific fungi and lack of activity against bacterial and animal cells^18^. Moreover, the selectivity of FE has been significantly influenced by the environmental lipid composition of target cell membranes. It has been confirmed by microbiological tests^19^ and studies performed using model systems^20^. The sensitivity of plant pathogens to FE was shown to correlate with the ergosterol (ERG) content, amount of charged lipids, and average length of the fatty acyl chains of lipids in the target cell membranes^19^.

The aggregation of FE on the membrane surface plays a key role in cell disruption. Heerklotz *et al*.^20^ showed that leakage of the fluorophore from lipid vesicles induced by FE was strongly inhibited by cholesterol as well as by palmitoyloleoylphosphatidylethanolamine (POPE) and palmitoyloleoylphosphoglycerol (POPG) lipids. The authors explain this result by reductions in FE lipid demixing and aggregation, which is believed to be required for membrane permeabilization. When ERG is present in the lipid composition, FE activity is weakly suppressed^20^. Recently, Mantil *et al*.^21^ showed that FE activity suppression by ERG might be related to the inhibition of the ordering effect of lipopeptide by sterol. Using molecular dynamics simulation, Sur *et al*.^22^ and Horn *et al*.^23^ showed that the probability of the formation of FE clusters in the membrane and their size depend on the interactions between lipopeptide molecules and certain lipid types, specifically palmitoylphosphatidylcholine (POPC), POPE, and POPG.

In the present study, the ability of FE to form ion-permeable pores in model lipid membranes mimicking the target fungal membranes and comprising POPC, POPE, POPG and ERG was extensively investigated. The properties of single FE-induced channels and methods of regulating the lipopeptide pore-forming activity were studied. To evaluate the role of lipid intrinsic curvature and lipid head group charge in the pore-forming activity of FE, planar bilayers with different lipid compositions, including glyceromonooleate (GMO), tetraoleoylcardiolipin (TOCL), and without POPE, have been used. To investigate whether membrane dipole potential and lipid packing influence the pore-forming activity of FE, low-molecular weight amphiphilic membrane modifiers, such as flavonoids, styryl dyes, thyroid hormones, detergents, local anesthetic, and alkaloids, were used.

## Results and Discussion

### The single FE channels

**Figure 2:**
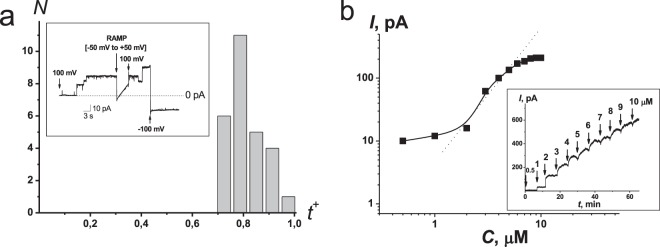
(**a**) Anion/cation selectivities of FE channels. A transfer number for potassium cations (*t*^+^) was determined at 10-fold concentration gradient of electrolyte (2 M KCl together with 10 mM CHES-KOH, pH 9 at the cis side and 0.2 M KCl at the *trans* side) across the bilayer composed of POPC:POPE:POPG:ERG (20:20:50:10 mol%) and modified with sufficient FE (*cis*-side only) to induce a bilayer conductance of 100–900 pS. *Inset*: Time course of the transmembrane current in voltage-changing experiments at 10-fold concentration gradient of electrolyte. The moments of voltage jumps and RAMP-application are marked by arrows. The voltage magnitudes are shown above the corresponding arrows. (**b**) Dependence of the steady-state FE-induced transmembrane current on the concentration of lipopeptide in bilogarithmic coordinates. The transmembrane voltage was 50 mV. *Insets*: The time courses during successive additives of FE are indicated by the arrows. The membrane was composed of POPC:POPE:POPG:ERG (20:20:50:10 mol%) and bathed in 2 M KCl and 10 mM CHES, pH 9.

**Figure 3:**
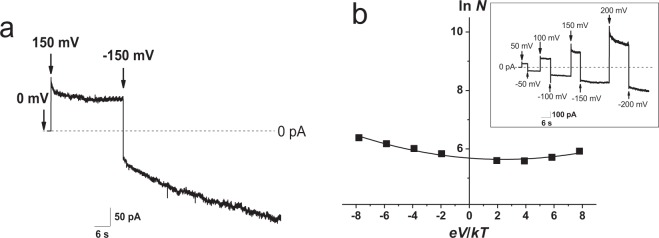
(**a**) Time course of the bilayer current in field-reversal experiments in the presence of FE in the bilayers. The time of voltage application is marked by arrows. The transmembrane potential was ±150 mV. (**b**) The natural logarithm of the steady-state number of open FE channels (ln*N*) in a lipid bilayer modified with 6 μM as a function of the dimensionless transmembrane voltage (*eV*/*kT*). The dependence is fitted by a polynomial of the second order. *Insets*: Time course of the bilayer current in field-reversal experiments in the presence of FE in the bilayers. The time of voltage application is marked by arrows. The membrane was composed of POPC:POPE:POPG:ERG (20:20:50:10 mol%) and bathed in 2 M KCl and 10 mM CHES, pH 9.

The effects of FE were examined in planar lipid bilayers composed of POPC:POPE:POPG:ERG and bathed in 2 M KCl (10 mM CHES-KOH, pH 9). The addition of FE at one (*cis*) side of the bilayer up to 0.1 to 0.5 μM in the membrane-bathing solution led to the appearance of step-like fluctuations of various amplitudes in the picoampère range. Figure 1a shows typical current traces recorded at ±150 mV. This result clearly indicates the ability of FE to form ion-permeable pores. The dwell time of FE pores did not exceed 250 ms, and the open probability was approximately 0.5. Figure 2S (Supplementary Information) summarizes the single-channel event statistics. The *I* value histograms demonstrate at least two different substates with close but distinguishable amplitudes. The observed heterogeneity of the channel population might be explained by the presence of two FE isoforms produced by the *Bacillus subtilis* strain F-29-3 (see Fig. 1S, Supplementary Information). FE A and FE B have only slight differences in the types of aliphatic amino acid residues that could affect pore size due to steric hindrance. On the other hand, the existence of two sublevels might be explained by the formation of different FE heteromers during pore formation or FE penetrating into the opposite monolayer. It should be noted that the probabilities of fluctuations with smaller and larger amplitudes were not the same, and the ratios of the probabilities depended on the sign of the applied voltage; at positive transmembrane potentials ranging from 100 to 200 mV, the probability of opening the channels with a larger amplitude was higher than with a smaller amplitude. The opposite situation was observed at negative voltages ranging from −200 to −100 mV. Figure 2S (Supplementary Information) shows the current transition histograms of single FE channels at ±150 and ±200 mV. The areas under the peaks characterize the probabilities of the current fluctuations of the corresponding amplitudes. Figure 3S (Supplementary Information) presents the dependence of the ratios of the probabilities of the functioning channels with larger and smaller amplitudes on the transmembrane voltage. The results indicate that FE channels, which were characterized by larger and smaller amplitudes at positive and negative transmembrane voltages, respectively, were most frequently observed. At a low absolute value voltage of ±50 mV, a transition state was probable. Figure 1b shows the dependence of the conductance of these channels on the transmembrane voltage. The conductance of the single channels was practically voltage-independent in the case of negative voltages up to −200 mV. For positive voltages, the conductance of the most often observed single channels slightly increased with voltage, increasing by approximately 1.4-fold from +50 to +200 mV. The asymmetric *g*(*V*) characteristic might indicate a nonuniform distribution of electric charge along the channel’s axis. More likely, the asymmetry of the *g*(*V*) curve of single FE channels might be related to the location of glutamic acid residues closer to the *cis* pore mouth than to the *trans* pore mouth. A lipopeptide from *Pseudomonas syringae* syringomycin E also forms ion channels characterized by an asymmetrical *g*(*V*) curve^24^. The increase in conductance of single syringomycin channels with negative potential was 1.4-fold higher than that of those with positive potential^25^. The asymmetry of *g*(*V*)-characteristic syringomycin channels is thought to be related to the positive net charge of lipopeptide molecules forming the *cis* pore mouth^26,27^. Thus, the mirror difference in the *g*(*V*) characteristics of syringomycin and FE channels is due to the opposite charges of lipopeptide molecules.

### Cation-anion selectivity of FE channels

The cation-anion selectivity of FE channels in POPC:POPE:POPG:ERG was determined for KCl. A potential of zero current (reversal potential) was measured after the formation of a 10-fold transmembrane concentration gradient of electrolyte (2 M KCl at the *cis* side and 0.2 M KCl at the *trans* side) across the bilayer modified with sufficient FE (*cis-*side only) to induce a bilayer conductance of 100–900 pS. The average reversal potential was about −35 mV. This value corresponds to weak cation selectivity. Figure 2a presents *t*^+^ histogram. The slight difference between the distribution obtained and the normal one might indicate the heterogeneity of the channel population, but the selectivity of substates (probably related to channels of low and high conductance) is practically of the same value and indistinguishable within the measurement error. The mean transfer number for cations is equal to 0.8 ± 0.1.

The most likely explanation for cation selectivity of both substates would be a high near-membrane concentration of cations created by a negative charge located at the entry of the channel. The sign of selectivity was consistent with the occurrence of negatively charged lipopeptide and lipid (namely, POPG) molecules. Fig. 6S (Supplementary Information) shows the plot of the mean reversal potential *vs*. the ratio of KCl activities in the bathing solution of *cis* and *trans* compartments in the logarithmic coordinates. The linearity of the dependence of the reversal potential on the ratio of KCl activities in the *cis* and *trans* compartments indicates that the ionic strength of the *cis* and *trans* solutions (changeable from 0.02 to 2 M KCl) did not significantly affect the selective filter of the FE-induced pore.

### Macroscopic conductance of FE treated membranes

The *cis*-side addition of FE to the POPC:POPE:POPG:ERG membrane-bathing solution over 1 μM increased the macroscopic membrane conductance in a dose-dependent manner. An example of a concentration/effect curve is presented in Fig. 2b. The figure shows a plot of the logarithm of the steady-state FE induced transmembrane current at *V* = 50 mV *vs* that of the lipopeptide concentration. The slope of the linear regression of the presented data is close to 2, as the current induced by FE enlarged with the second power of the lipopeptide concentration. This result suggests the oligomerization of FE molecules during the formation of pores, and at least a dimer is needed to form the channel. A similar situation occurs in the case of another lipopeptide produced by *Bacillus subtilis*, surfactin; the macroscopic lipopeptide-induced conductance enhances with a slope of 2 on a bilogariphmic plot as a function of the surfactin concentration. These data showed that surfactin dimers were involved in the functional channel formation process^14^.

**Figure 4:**
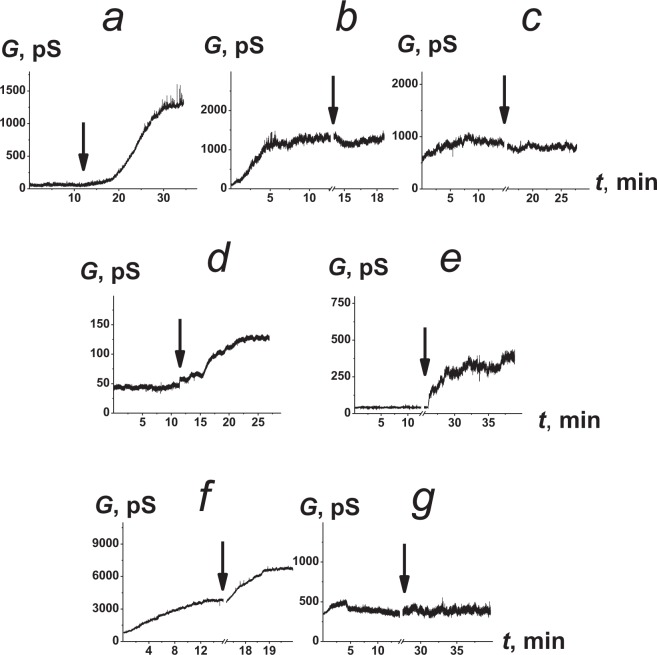
The effects of different modifiers of the electrical and elastic properties of the tested membranes on the steady-state bilayer conductance induced by FE. The moments that the modifiers (20 μM phloretin (**a**), 5 μM RH 421 (**b**), 20 μM myricetin (**c**), 1 mM TTC (**d**), 50 μM T_3_ (**e**), 10 μM TX-100 (**f**), and 1 mM caffeine (**g**)) were added to the bilayer bathing solution are indicated by the arrows. The transmembrane voltage was 50 mV. The membranes were composed of POPC:POPE:POPG:ERG (20:20:50:10 mol%) and bathed in 2 M KCl and 10 mM CHES, pH 9.

The time course of the transbilayer current in response to an initial positive transmembrane voltage step followed by a negative voltage step (from +150 to −150 mV) in the POPC:POPE:POPG:ERG bilayer modified by the *cis*-side addition of FE is depicted in Fig. 3a. At a positive voltage, the current decreased as a double exponential function. The characteristic times of the fast and slow components were approximately 0.5 and 6 sec, respectively. An abrupt *V*-change from +150 to −150 mV led to an instantaneous 1.3-fold decrease in the absolute magnitude of the FE-induced conductance and exponential current growth with the characteristic time of approximately 50 sec. The number of channels cannot change instantaneously and therefore the sharp decrease in the current amplitude was most likely related to the abovementioned difference in single-channel conductance, as the conductance ratio of single FE channels at 150 and −150 mV is about 1.5 (Fig. 1b). As single channels of the larger and lower conductance were preferentially observed at high positive and negative potentials (at |*V*| ≥100 mV), respectively (Fig. 2S, Supplementary Information)), one can propose that transitions of FE channels from the larger conductance state to the lower amplitude proceeded instantaneously. The relaxation processes followed the voltage sign switching also indicate the different ability of FE channel substates to respond to the application of voltages of different signs. The slow current growth at negative voltages was due to consistent channel openings. In addition, the association/aggregation of FE channels of low amplitudes might occur at negative voltage. This assumption does not contradict the data on the production of lipopeptide-rich domains within the bilayer by low concentrations of FE^28,29^. The latter phenomenon might result in subsequent membrane disintegration. These data are in agreement with the observation that the threshold of the electrical stability loss of the lipid bilayer is on average 1.3 times less when negative potentials are applied than when positive transmembrane voltages are applied. Figure 4S (Supplementary Information) clearly demonstrates that the sequence of voltage switching (from positive to negative or vice versa) did not affect the voltage gating nature of FE channel openings and closings. A comparison of Figs 3a and 4S shows that the decrease in the absolute transmembrane voltage value (from 150 to 100 mV) slowed the kinetics of both pore closing and opening. It should be noted that at potentials of absolute values less than 100 mV, the effective number of channels functioning in the membrane remained almost unchanged during the observation period (several minutes).

The dependence of the stationary conductance of FE treated bilayers on transmembrane voltage was also studied. The effective number of channels, *N*_*ch*_(*V*), was calculated as the ratio of the quasi-steady-state bilayer conductance to the amplitude of most frequently observed channels (presented in Fig. 1b) at *V* = const. The contributions of different channel conductance substates to macroscopic FE-induced conductance were not taken into account. The plot of the natural logarithm of the effective number of open pores on the applied dimensionless transbilayer voltage (*eV*/*kT*) is presented in Fig. 3b. The mean number of open pores under the steady-state conditions, *N*_*ch*_(*V*), is concerned to the excess channel formation work, *W*_*ch*_ = *W*_*ch*_(*V*) − *W*_*ch*_(0), as determined by the Boltzmann distribution^30^:

1 $${N}_{ch}\,(V)=\,{N}_{ch}(0)\exp (\frac{{W}_{ch}(0)-{W}_{ch}(V)}{kT})\,$$

where *N*_*ch*_(0), the number of channels at *V* = 0, is a constant owing to equilibrium between the precursors of the channels in the membrane and FE molecules in the bathing solution, *k* is a Boltzmann constant, and *T* is an absolute temperature.

The channel formation work *W*_*ch*_(*V*) is composed of the structural component, *W*_*ch*_(0), the Coulomb component, which is equal to the product of the effective gating charge, *q*, elementary charge, *e*, and *V* and related to the interaction of charged and dipole gating particles in FE channel with the intramembrane electric field, and an additional component which is proportional to the voltage squared and concerned to the possible electrostriction

2 $${W}_{ch}(V)={W}_{ch}\,(0)\,\,-\,eqV\,-\,\alpha {V}^{2}$$

where α is a constant related to electrostriction^31^.

This equation leads to

3 $${N}_{ch}\,(V)=\,{N}_{ch}(0)\exp (\frac{eqV\,+\,\alpha {V}^{2}}{kT})\,$$

or

4 $$\mathrm{ln}\,{N}_{ch}\,(V)=\,\mathrm{ln}\,{N}_{ch}(0)+q\frac{eV}{kT}\,+\,\alpha ^{\prime} {(\frac{eV}{kT})}^{2}$$

where α′ is an electrostriction constant at the transition to a dimensionless transmembrane potential

$$(\alpha ^{\prime} =\alpha \frac{kT}{{e}^{2}})$$

Fitting the data presented in Fig. 3b with a second order polynomial function elucidates that *N*_*ch*_(0), *q*, and α′ are 300 ± 5, −0.037 ± 0.004, and 0.008 ± 0.001, respectively. Thus, voltage gating and electrostriction force make minor but notable contributions to the work of pore formation in membranes treated with FE.

### Channel-forming activity of FE: effects of the spontaneous curvature, lipid charge, membrane dipole potential, and lipid packing

Molecular dynamics simulation performed by Horn *et al*.^23^ revealed that FE promotes membrane positive curvature. Based on these data, one can hypothesize that the shape of lipid molecules plays a significant role in the pore-forming activity of the lipopeptide. To test this idea, we use TOCL, a negatively charged lipid possessing four acyl chains and thereby inducing high negative curvature stress^32^. The coefficient of FE binding with the membranes composed of POPC:POPE:TOCL:ERG (40:40:10:10 mol%) was equal to 2 (Fig. 5Sa, Supplementary Information), as was the case for POPC:POPE:POPG:ERG bilayers (Fig. 2b). Thus, replacement of a lipid with a conical shape (POPG) with that with an inverted conical shape (TOCL) did not significantly alter the pore-forming activity of FE. In addition, the decrease in the negative curvature stress due to complete removal of POPE, the neutral species, having an inverted conical shape similar to that of TOCL did not affect the ability of FE to form pores. The coefficient of cooperative binding of FE to POPE-free lipid membranes composed of POPC:POPG:ERG (40:50:10 mol%) was also approximately 2 (Fig. 5Sb, Supplementary Information). The data obtained might indicate that the FE channels are not characterized by pronounced positive curvature.

In the literature, evidence of the dependence of FE activity on the presence of negatively charged species in the membrane can be found^33^. To estimate the contribution of lipid charge to the channel formation ability of FE, we investigated the effects of replacing the negatively charged POPG with uncharged GMO. The *cis*-side addition of FE to the POPC:POPE:GMO:ERG (20:20:50:10 mol%) bilayer bathing solution did not increase the membrane conductance. One can conclude that the presence of negatively charged lipids is absolutely required for pore formation by FE. Two explanations for this result are possible: electrostatic repulsion occured between negatively charged lipid heads promotes the immersion of lipopeptide molecules into the bilayer or electrostatic repulsion is responsible for the “correct” folding of the FE molecule peptide ring.

To test the first assumption, we used modifiers of the membrane dipole potential (φ_*d*_). φ_*d*_ is a component of the electric potential drop at the membrane-solution interface related to lipid headgroups and water dipoles. The membrane dipole potential substantially affects the embedment of syringomycin E and surfactin into lipid bilayers^34–36^. Figure 4 (a–e) demonstrates the effects of various dipole modifiers on the macroscopic conductance induced by FE in the POPC:POPE:POPG:ERG membranes. Phloretin, TTC, and T_3_ increased the steady-state FE-produced membrane conductance (Fig. 4a,d,e), while the introduction of myricetin and RH 421 (Fig. 4b,c) did not practically affect the lipopeptide multichannel activity. Table 1 presents the mean ratios of the FE-induced steady-state membrane conductance in the presence and absence of the tested modifiers (*G*_*modifier*_*/G*_*control*_). The addition of phloretin, T_3_, and TTC led to 4-5-fold increases in the steady-state membrane conductance induced by FE. The mean *G*_*modifier*_*/G*_*control*_ values for myricetin and RH 421 were approximately 1 (Table 1). We measured the changes in the φ_*d*_ of POPC:POPE:POPG:ERG membranes produced by the tested modifiers. Table 1 shows that the reduction in φ_*d*_ due to the adsorption of phloretin and T_3_ was approximately 70 and 60 mV, respectively. The addition of RH 421 and TTC to the membrane-bathing solutions led to approximately 90 and 80 mV increases in φ_*d*,_ respectively. Table 1 also shows that myricetin did not practically affect the φ_*d*_ value of POPC:POPE:POPG:ERG bilayers. Comparing the effects of well-known dipole modifiers on the dipole potential of POPC:POPE:POPG:ERG membranes and channel-forming activity of FE, one can conclude that the observed effects of the tested modifiers on the multichannel activity of FE are not related to the membrane dipole potential. Thus, the results obtained contradict the assumption that significant FE molecule immersion into the bilayer occurs during pore formation. Otherwise, the channel-forming activity of the negatively charged FE would depend on the electric potential jump between the aqueous solution and the hydrocarbon interior.

**Table 1:** Characteristic parameters of the effects of FE on the properties of lipid bilayers: *G*_*modifier*_*/G*_*control*_ – ratio of the membrane conductance of FE channels in the absence and presence of membrane modifiers at *V* = ±50 mV; Δ*φ*_b_ – change in the membrane boundary potential related to its dipole component; Δ*T*_*m*_ – change in the main transition temperature of DPPC after the addition of modifier to the suspension of liposomes; Δ*T*_*1/2*_ – change in the half-width of the peak.

*modifiers*	C, μM	*G*_*modifier*_ */G*_*control*_^a^	Δ*φ*_b_, mV^b^	−Δ*T*_*m*_, °C	Δ*T*_*1/2*_, °C
*Phloretin*	20	3.8 ± 1.6	−70 ± 2	1.2	1.9
*Myricetin*	20	1.0 ± 0.1	5 ± 2	0.1	0.2
*T*_*3*_	50	4.7 ± 1.5	−60 ± 8	0.8^#^	0.2
*RH 421*	5	1.3 ± 0.2	90 ± 15	0.1	0.2
*TTC*	1000	5.4 ± 2.1	80 ± 12	3.5^$^	0.7
*TX-100*	10	2.1 ± 0.5	1 ± 1	2.4	1.6
*Caffeine*	1000	1.0 ± 0.1	3 ± 1	0	0

^a^The membranes were composed of POPC:POPE:POPG:ERG (20:20:50:10 mol%) and bathed in 2 M KCl and 10 mM CHES, pH 9.

^b^The POPC:POPE:POPG:ERG-membranes bathed in 0.1 M KCl and 10 mM CHES, pH 9.

^#^The results by^30^.

^$^The results by^41^.

According to the literature, dipole membrane modifiers can affect not only the electrical but also the elastic properties of the membrane, namely, the lipid packing density^30,37–41^. Changes in the packing density of membrane lipids can be detected by studying lipid phase transitions. Membrane disordering is accompanied by a decrease in the temperature of the main phase transition (Δ*T*_*m*_) and a decrease in its cooperativity. Transition cooperativity can be estimated by the half-width of the main peak (Δ*T*_*1/2*_), and higher peak half-widths are associated with less cooperativity of lipid transition from one phase state to another. Table 1 presents the Δ*T*_*m*_ and Δ*T*_*1/2*_ values produced by the addition of the tested modifiers to the DPPC liposome bathing solution. Phloretin, T_3_, and TTC significantly affected the lipid phase behavior, while myricetin, and RH 421 did not practically change DPPC melting. On this basis, one can assume that the increase in *G*_*modifier*_*/G*_*control*_ in the presence of phloretin, T_3_, and TTC occurred due to a decrease in the packing density of lipids in the membrane. To confirm this assumption, we used an agent with a well-known disordering action, TX-100^42^. As a negative control, we used alkaloid caffeine, which does not cause such changes in the packaging of lipids in the bilayer^43^. Figure 4 demonstrates the effects of TX-100 (f) and caffeine (g) on the macroscopic conductance induced by FE in the POPC:POPE:POPG:ERG membranes. As expected, TX-100 potentiated the pore-forming activity of FE, while caffeine did not affect this activity. Thus, the increase in *G*_*modifier*_*/G*_*control*_ at the adsorption of phloretin, T_3_, TTC, and TX-100 correlates with the decreased membrane lipid packing density that potentially contributes to peptide oligomerization. These data are consistent to that of Mantil *et al*.^21^, which showed that the microbial sensitivity to FE correlates with a greater fluidity of membranes of target microorganisms compared to those of tolerant microorganisms.

Dose-dependence of FE induced macroscopic membrane conductance in bilayers composed of dioleoylphosphocholine:cholesterol (67:33 mol%) and POPE:POPG (50:50 mol%) mimicking bacterial and mammalian cell membranes respectively has been additionally examined. FE adsorption did not practically affect the conductance of these bilayers. Taking into account the low hemolytic activity of FE, the lack of evidence for its bactericidal properties and the promising antifungal action of lipopeptide^2,3,8,11^ one may conclude that pore forming hypothesis well explains the specific biological activity of the FE.

## Conclusions

Using the model lipid membranes, we demonstrated that the lipopeptide from *Bacillus subtilis* FE forms ion channels of weak cation selectivity in lipid bilayers specifically mimicking the composition of target fungal cell membranes. The FE-induced conductance enlarged with the second power of the FE aqueous concentration, indicating that at least dimers are required for pore formation. A voltage switching from a positive to a negative value led to (i) an abrupt decrease in the amplitude of single channels that occurred simultaneously with (ii) an exponential increase in steady-state transmembrane conductance over time due to channel openings. The dependence of the steady-state FE-induced macroscopic current on *V* revealed that voltage gating and electrostriction force make minor but notable contributions to pore formation in membranes treated with FE. The negatively charged lipids contribute to pore formation by FE, which should be related to the folding of the lipopeptide molecule in the bilayer.

## Materials and Methods

Lipids were purchased from Avanti Polar Lipids, Inc. (Pelham, AL): 1-palmitoyl-2-oleoyl-*sn*-glycero-3-phosphocholine (POPC), 1-palmitoyl-2-oleoyl-*sn*-glycero-3-phosphoethanolamine (POPE), 1-palmitoyl-2-oleoyl-*sn*-glycero-3-phospho-(1’-rac-glycerol) (POPG), 1,1′,2,2′-tetraoleoyl cardiolipin[4-(dipyrrometheneboron difluoride)butanoyl] (ammonium salt) (TOCL), 1-oleoyl-rac-glycerol (GMO), 1,2-dipalmitoyl-*sn*-glycero-3-phosphocholine (DPPC), and ergosterol (ERG) Phloretin, myricetin, 3,3′,5-triiodo-L-thyronine (T_3_), 4-{4-[4-(dipentylamino)phenyl]-1,3-butadienyl}-1-(4-sulfobutyl)pyridinium hydroxide (RH 421), tetracaine hydrochloride (TTC), Triton X-100 (TX-100), caffeine, nonactin, KCl, CHES, HEPES, KOH, and hexadecane were purchased from Sigma Chemical (St. Louis, MO).

Fengycin, purchased from Sigma Chemical, is a lipopeptide mixture synthesized by the *Bacillus subtilis* strain F-29-3. It is composed of two components, FE A and FE B (Fig. 1S, Supplementary Information).

### Detection of lipopeptide induced currents flowing through the planar lipid bilayers: single pores and macroscopic conductance

Using a monolayer opposition technique the virtually solvent-free lipid bilayers were made^44^ on a 50-100-μm-diameter aperture and a 10-μm-thick Teflon film separating two (*cis* and *trans*) Teflon chamber compartments. The aperture was pretreated with hexadecane. To specifically imitate the target fungal cell membranes, model membranes were prepared from mixtures of POPC:POPE:POPG:ERG (20:20:50:10 mol%)^19^. To test the lipid specificity of FE, the membranes were also prepared from mixtures of POPC:POPE:GMO:ERG (20:20:50:10 mol%), POPC:POPE:TOCL:ERG (40:40:10:10 mol%), and POPC:POPG:ERG (40:50:10 mol%). The 2 M KCl solutions were the same in the *cis* and *trans* Teflon chamber compartments and were buffered by 10 mM CHES-KOH at pH 9. A net charge of FE is expected to be strongly negative at pH 9.0 (pKa values of negatively ionizable side-chain groups in aqueous solution are 4.5 for two glutamic acid residues^45^), and the electrostatic repulsions between FE molecules should prevent aggregate formation in the bathing solution. After the lipid bilayer was completely formed and stabilized, lipopeptide from a stock solution (0.7 mM in methanol) was added to the bathing solution at the (*cis*)-side of the membrane to obtain a final concentration ranging from 0.5 to 10 μM. The upper limit was close to the critical micelle concentration of FE, which is approximately 11 mg/l in 5 mM Tris (pH 8, 20 °C, 7.5 μM)^46^.

To clamp the transbilayer voltage (*V*) and assess the current flowing through the membrane (*I*) Ag/AgCl electrodes with 1.5% agarose/2 M KCl bridges were used. Positive sign of transmembrane voltage means that the *cis* chamber compartment is positive relative to the *trans* one.

The recording of *I* were carried out at room temperature in the voltage-clamp mode using an Axopatch 200B amplifier and Digidata 1440 A and analyzed with pClamp 10 (Molecular Devices, Orlean, CA, USA) and Origin 8.0 (OriginLab Corporation, Northampton, MA, USA). A frequencies of sampling and low-pass filtering were equal to 5 and 200 Hz, respectively. The current tracks were processed through an 8-pole Bessel 100-kHz filter.

The *I* values histograms were made for the tested transmembrane voltages. The total number of channel fluctuations (*N*) used for statistical analysis ranged from 300 to 900. Peaks on the current-transition histograms were fitted by the sum of normal density functions. The χ^2^ criterion was applied (*P* < 0.05).

A steady-state FE-induced transmembrane current (*I*_*∞*_) was used to assess the channel-forming activity of lipopeptide after and before two-sided additions of a modifier (phloretin, myricetin, RH 421, TX-100, TTC, T_3_, and caffeine). The mean ratios (*I*_*∞*_*/I*_*∞*_^0^) of macroscopic conductances after (*I*_*∞*_) and before (*I*_*∞*_^0^) two-sided modifier addition were averaged from 3 to 9 bilayers (mean ± SE).

The modifiers were added at both sides of the membranes up to 20 μM (phloretin and myricetin), 5 μM (RH 421), 10 μM (TX-100), 1 mM (TTC and caffeine), and 50 μM (T_3_).

### Cation-anion selectivity of membranes treated with fengycin

The transfer numbers for potassium cations (*t*^+^) and chlorine anions (*t*^*−*^ = 1 − *t*^+^) were evaluated by measuring the reversal potential (*V*^*rev*^) under different salt concentration gradients and by using the general expression^47^

5 $${V}^{rev}=(kT/e)(1-2{t}^{+}){\rm{l}}{\rm{n}}({\gamma }_{1}{C}_{1}/{\gamma }_{2}{C}_{2})$$

where *γ*_1_ and *γ*_2_ indicate the activity coefficients, *C*_1_ and *C*_2_ are KCl concentrations in the *cis* and *trans* compartment, respectively.

The membranes were composed of POPC:POPE:POPG:ERG (20:20:50:10 mol%) and bathed in initially separated asymmetric salt solutions of 0.02 M KCl (*trans*) or 2 M KCl (*cis*) together with 10 mM CHES-KOH, pH 9. The KC1 concentration in the *trans* compartment was increased by adding aliquots of 4 M KCl and 10 mM CHES-KOH, pH 9.

### Measurement of boundary potential at the membrane/solution interface

The steady-state transmembrane current induced by K^+^-nonactin was modulated via the two-sided addition of 20 μM phloretin and myricetin (stock solution 40 mM in ethanol), 5 μM RH 421 (stock solution 10 mM in ethanol), 50 μM for T_3_ (stock solution 20 mM in DMSO), 10 μM for TX-100 (stock solution 100 mM in water), 1 mM for TTC (stock solution 300 mM in water) and 1 mM for caffeine (stock solution 75 mM in water) into the membrane-bathing solution (0.1 M KCl, 10 mM CHES-KOH, pH 9). The lipid bilayer were made from POPC:POPE:POPG:ERG (20:20:50:10 mol%). The FE-induced membrane conductance was determined by measuring *I* at a voltage clamp condition (*V* = 50 mV). The subsequent calculations were performed assuming that the bilayer conductance was related to the bilayer boundary potential, the potential drop between the aqueous solution and the bilayer hydrophobic core (φ_*b*_), by the Boltzmann distribution^48^:

6 $$\Delta {\varphi }_{b}=(kT/e)\mathrm{ln}\,({G}_{m}/{G}_{m}^{0})$$

where *G*_*m*_ and ${G}_{m}^{0}$ are the steady-state bilayer conductance induced by nonactin in the presence and absence of modifiers, respectively, and *e*, *k* and *T* have their usual meanings. The mean values of Δφ_*b*_ were averaged from 3 to 5 model membranes (mean ± SD).

### Differential scanning calorimetry measurements

Differential scanning calorimetry experiments were performed using a μDSC 7EVO microcalorimeter (Setaram, France). Large unilamellar liposomes were made from DPPC by the electroformation method (3 V, 10 Hz, 1 h, 55 °C). The suspension of vesicles contained 5 mM lipid and was buffered by 5 mM HEPES-KOH at pH 7.4. Modifiers were added to the aliquots up to 20 μM phloretin, 20 μM myricetin, 5 μM RH 421, 10 μM TX-100, and 1 mM caffeine. The liposomal suspension was heated with a rate of 0.2 K·min^−1^. The reversibility of the thermal transitions was assessed by reheating the sample immediately after the cooling step from the previous scan. Using Calisto Processing (Setaram, France) the temperature dependence of the excess heat capacity was analyzed. The pre-transition and the main transition (*T*_*m*_) of DPPC were observed at 34.1 °C and 41.3 °C. The half-width of the main peak (*T*_*1/2*_) was about 0.5 °C.

## Supplementary information

Supplementary Information

## Data Availability

The sufficient datasets are available on a reasonable request to the corresponding author.
