# Supplementary material for: Fengycin induces ion channels in lipid bilayers mimicking target fungal cell membranes
Source: Sci Rep. 2019 Nov 5;9:16034. doi: 10.1038/s41598-019-52551-5 (PMC6831686; doi:10.1038/s41598-019-52551-5)
Supplement: Supplementary file 1 — Supplementary Information [file 41598_2019_52551_MOESM1_ESM.docx]

**Fengycin induces ion channels in lipid bilayers mimicking target fungal cell membranes**

*Anastasiia A. Zakharova^1^*^,§^*, Svetlana S. Efimova^1,^*^§,^**, Valery V. Malev^1,2^, Olga S. Ostroumova^1^*

*^1^* Institute of Cytology of the Russian Academy of Sciences, St. Petersburg, 194064

*^2^* St. Petersburg State University, Petergof, 198504, Russia

*^§^Anastasiia Zakharova and Svetlana Efimova contributed equally to this work.*

*^*^Correspondence and requests for materials should be addressed to S.S. (email: efimova@incras.ru)*

**Supplementary Information**


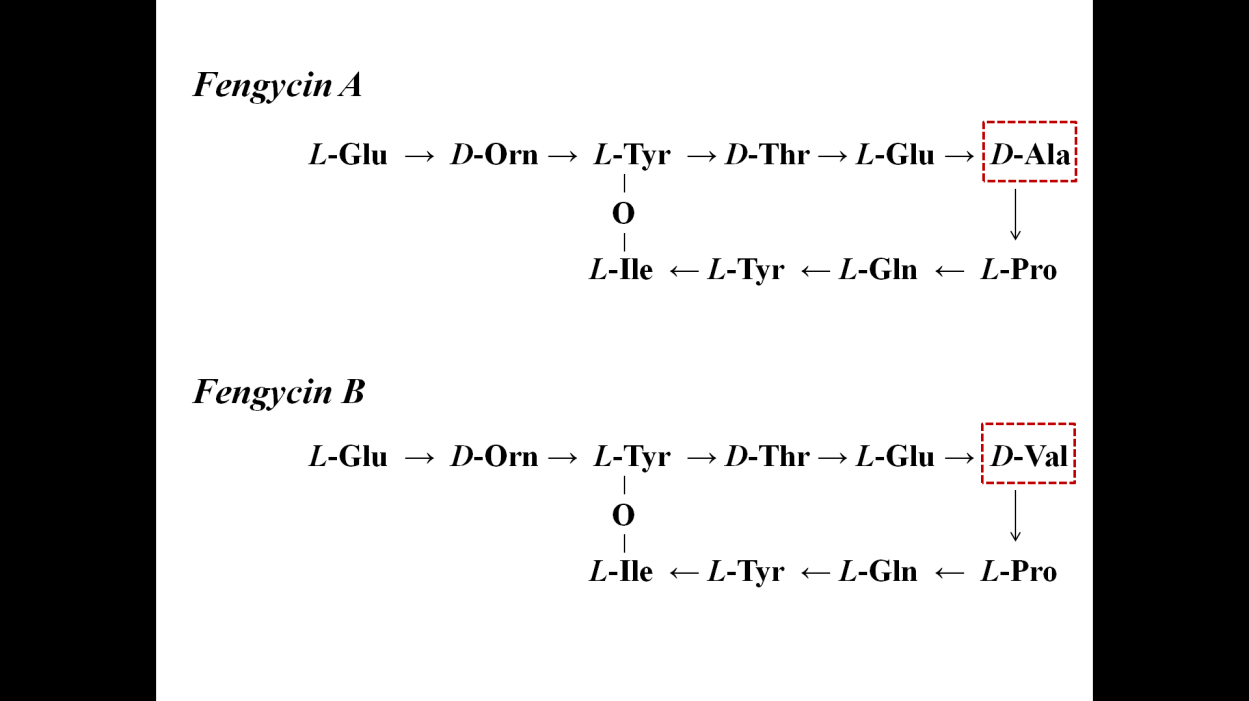


**Figure 1S.** The chemical structure of the FEs. The red rectangles indicate the fragments that differ in the FE peptide regions.

**Figure 2S.** Current-transition histograms of single FE channels at transmembrane voltages of 150 (**a**), -150 (**b**), 200 (**c**) and -200 mV (**d**). The membranes were composed of POPC:POPE:POPG:ERG (20:20:50:10 mol%) and bathed in 2 M KCl and 10 mM CHES, pH 9.

**Figure 3S.** Dependence of the ratios of probabilities of the opening channels with larger (ρ_high_) and smaller (ρ_low_) amplitudes on transmembrane voltage. The membranes were composed of POPC:POPE:POPG:ERG (20:20:50:10 mol%) and bathed in 2 M KCl and 10 mM CHES, pH 9.

**Figure 4S.** Time course of the bilayer current in field-reversal experiments in the presence of FE in the bilayers. The time of voltage application is marked by arrows. The transmembrane potential was ±100 mV. The membrane was composed of POPC:POPE:POPG:ERG (20:20:50:10 mol%) and bathed in 2 M KCl and 10 mM CHES, pH 9.

**Figure 5S.** Dependence of the steady-state transmembrane current induced by FE on the concentration of lipopeptide in bilogarithmic coordinates. The membranes were composed of POPC:POPE:TOCL:erg (40:40:10:10 mol%) (**a**) or POPC:POPG:erg (40:50:10 mol%) (**b**) and bathed in 2 M KCl and 10 mM CHES, pH 9. The transmembrane voltage was 50 mV. *Insets*: The time courses during successive additives of FE are indicated by the arrows.

**Figure 6S.** The reversal membrane potential as a function of the logarithmic ratio of KCl activities (*γC*) in the *cis* and *tran*s compartments. FE was added to the *cis* aqueous compartment at concentrations up to 2 µM. The membranes were composed of POPC:POPE:POPG:ERG (20:20:50:10 mol%), initially separated by asymmetric salt solutions of 0.02 M (*trans*) and 2 M KCl (*cis*) together with 10 mM CHES-KOH, pH 9. The KC1 concentration in the *trans* compartment was increased by adding different aliquots of 4 M KCl and 10 mM CHES-KOH, pH 9 to that compartment.
